# P-1131. Reducing Surgical Site Infections: Optimizing Antibiotic Selection and Dosing for Surgical Prophylaxis

**DOI:** 10.1093/ofid/ofaf695.1325

**Published:** 2026-01-11

**Authors:** Punit J Shah, Amruta Mardikar, Gordana Isajloska Jasmak, Rajan Desai, Firas Zabaneh, Karl Nazareth

**Affiliations:** Houston Methodist Sugar Land Hospital, Sugar Land, TX; Houston Methodist Sugar Land Hospital, Sugar Land, TX; Houston Methodist Sugar Land Hospital, Sugar Land, TX; Houston Methodist Sugar Land Hospital, Sugar Land, TX; Houston Methodist, Houston, Texas; Houston Methodist Sugar Land Hospital, Sugar Land, TX

## Abstract

**Background:**

Surgical site infections (SSIs) contribute to increased healthcare costs, hospital lengths of stay, mortality, and morbidity. Antibiotic prophylaxis is standard for many surgeries to prevent SSIs. We evaluated the impact of pharmacists’ interventions on optimal preoperative antibiotic selection and dosing for surgical prophylaxis.

**Methods:**

A quasi-experimental study was conducted on patients that underwent an orthopedic surgical procedure. Patients in the pre-intervention group had a procedure between October – December 2023, and patients in the intervention group had a procedure between October – December 2024. In the intervention phase, a pharmacist reviewed patients undergoing a planned orthopedic procedure and optimized antibiotic selection and dosing according to institution guidelines for surgical prophylaxis approved by the Pharmacy and Therapeutics committee. Cefazolin was the first line agent prescribed for surgical prophylaxis and vancomycin was the alternative in a patient with severe penicillin allergy. A pharmacist contacted surgeons to improve antibiotic selection for any deviations. The primary outcome was the percentage of patients with optimal preoperative antibiotic selection (cefazolin or vancomycin for patients with severe penicillin allergy) and percentage of patients with optimal preoperative antibiotic dosing. The secondary outcome was the percentage of patients with surgical site infections for knee, hip, and spine procedures.

**Results:**

A total of 1,512 patients were included; 726 patients in the pre-intervention group and 786 patients in the intervention group. Pharmacist interventions significantly improved preoperative antibiotic selection, 99.1% (779/786) vs 97.4% (707/726) [95% CI -0.03,-0.004; p=0.016] and preoperative antibiotic dosing, 98.2% (772/786) vs 88.7% (644/726) [95% CI -0.12,-0.07; p=< 0.001]. Surgical site infections for knee, hip and spine procedures were also significantly lower in the intervention group, 1.4% (5/350) vs 4.5% (12/268) [95% CI 0.003, 0.06; p=0.026].

**Conclusion:**

Multi-disciplinary collaboration and pharmacist oversight help improve antibiotic dosing and selection for surgical prophylaxis. Optimizing prescribing practices is associated with a reduction in surgical site infections.

**Disclosures:**

All Authors: No reported disclosures

